# Younger Dryas glacier advances in the tropical Andes driven by increased precipitation

**DOI:** 10.1038/s41598-025-16603-3

**Published:** 2025-08-22

**Authors:** Neil F. Glasser, Stephan Harrison, Ryan Wilson, Joanne Wood, Matthew Peacey, Dylan Rood, Keir Nichols, Renato R. Colucci, Costanza Del Gobbo, Andrea Securo, J. C. Torres, Christian Riveros, Harrinson W. Jara, Enver Melgarejo, Hilbert Villafane, Manuel Cosi

**Affiliations:** 1 Department of Geography and Earth Sciences, Aberystwyth University, Wales, SY23 3DB UK; 2 Faculty of Environment, Science and Economy, Exeter University, Exeter, TR10 9EZ UK; 3 Division of Geography, University of Huddersfield, Huddersfield, UK; 4 Department of Earth Science and Engineering, Royal School of Mines, Imperial College London, London, SW7 2AZ UK; 5 Scottish Universities Environmental Research Centre (SUERC), University of Glasgow, East Kilbride, G75 0QF UK; 6 Institute of Polar Sciences, CNR, Trieste, Italy; 7 Department of Earth and Atmospheric Sciences, University of Quebec in Montreal (UQAM), Montreal, QC H3C 3P8 Canada; 8 Cà Foscari University in Venice, Venice, Italy; 9 Instituto Nacional de Investigación en Glaciares y Ecosistemas de Montaña (National Institute of Research on Glaciers and Mountain Ecosystems), Huaraz, Perú

**Keywords:** Climate sciences, Environmental sciences

## Abstract

There is currently a debate about the timing and drivers of former glacier behaviour and climate change in the tropical Andes. Using ^10^Be dating we determined the ages of 21 boulders on moraines in the Santa Cruz Valley, Peru (∼10°S, altitudes ~ 4100 to ~ 4300 m a.s.l.). Former glacier extent is marked by a suite of nested outer lateral and terminal moraines. These moraines are dated to 11.1 ka, 11.6 ka, 11.8 ka and 12.0 ka, falling within the Younger Dryas Chronozone (YDC; ∼12.9–11.6 ka). Nine ^10^Be samples from the Lake Arhuaycocha catchment document a period of glacier thinning and lateral contraction between 12.0 ka and 11.8 ka. Reconstructed glacier Equilibrium Line Altitudes (ELA) at 11.0 to 12.0 ka with an area–altitude balance ratio (AABR) of 1.00-2.50 are between 4675 and 4835 m a.s.l. for the Arhuaycocha glacier, between 4692 and 4832 m a.s.l. for the Taullicocha glacier and between 4800 and 4940 m a.s.l. for the Artizon glacier. These values represent a depression of 300–400 m in elevation compared to contemporary values for the ELA. We infer that the glacier advances at this time were driven by increased precipitation and that these changes were most likely a response to seasonal changes in the position of the ITCZ.

**Supplementary Information:**

The online version contains supplementary material available at 10.1038/s41598-025-16603-3.

## Introduction

Tropical glaciers are currently thinning and receding rapidly as a result of regional changes in temperature and precipitation^1,2^. These glaciers are important indicators of recent and past climate change^3,4^. Their past behaviour and response to the rapid climate changes following the global Last Glacial Maximum (gLGM) provides crucial insight into the spatial extent of the subsequent Antarctic Cold Reversal (ACR) and Younger Dryas Chronozone (YDC) cooling events. Previous studies^5^ have argued that glaciers in the northern tropics advanced during the ACR and then retreated during YD times in response to regional warming and reduced precipitation. Accurately dated moraine sequences are required to assess this assertion.

The tropical Andes are influenced by tropical Pacific and Atlantic ocean-atmospheric interactions and are a key location globally for understanding these dynamics^6,7^. The most critical climatic region is the location of the Intertropical Convergence Zone (ITCZ), a belt of low pressure that migrates seasonally between the northern and southern hemispheres, bringing with it heavy precipitation^8,9^. In the tropical Andes, changes in the position of the ITCZ affect the timing and amount of precipitation, which is critical for driving glacier behaviour^10^. During the wet season, when the ITCZ is typically positioned close to the equator, increased precipitation in the form of snow helps maintain positive mass balance for regional glaciers^11^. Given the sensitivity of glaciers to changes precipitation in the Tropical Andes, it is likely that any periods of advance will have been influenced by this parameter and the positionality of the ITCZ.

Palaeoglaciological evidence in the form of lateral and terminal moraines provides fundamental information with which to reconstruct former glacier positions and therefore climate variability over glacial-interglacial timescales. Mapping of these landforms, when coupled with geochronology, provides insights into former glacier dynamics, behaviours and extent at specific times and locations^12^. Unlike many glaciers further south in South America, tropical Andean mountain glaciers have never undergone lacustrine calving or developed extensive debris-covered surfaces, both of which serve to obscure the glacier - climate signal. Their past behaviour therefore gives us a relatively clear view of glacier response to climate change.

**Fig. 1:**
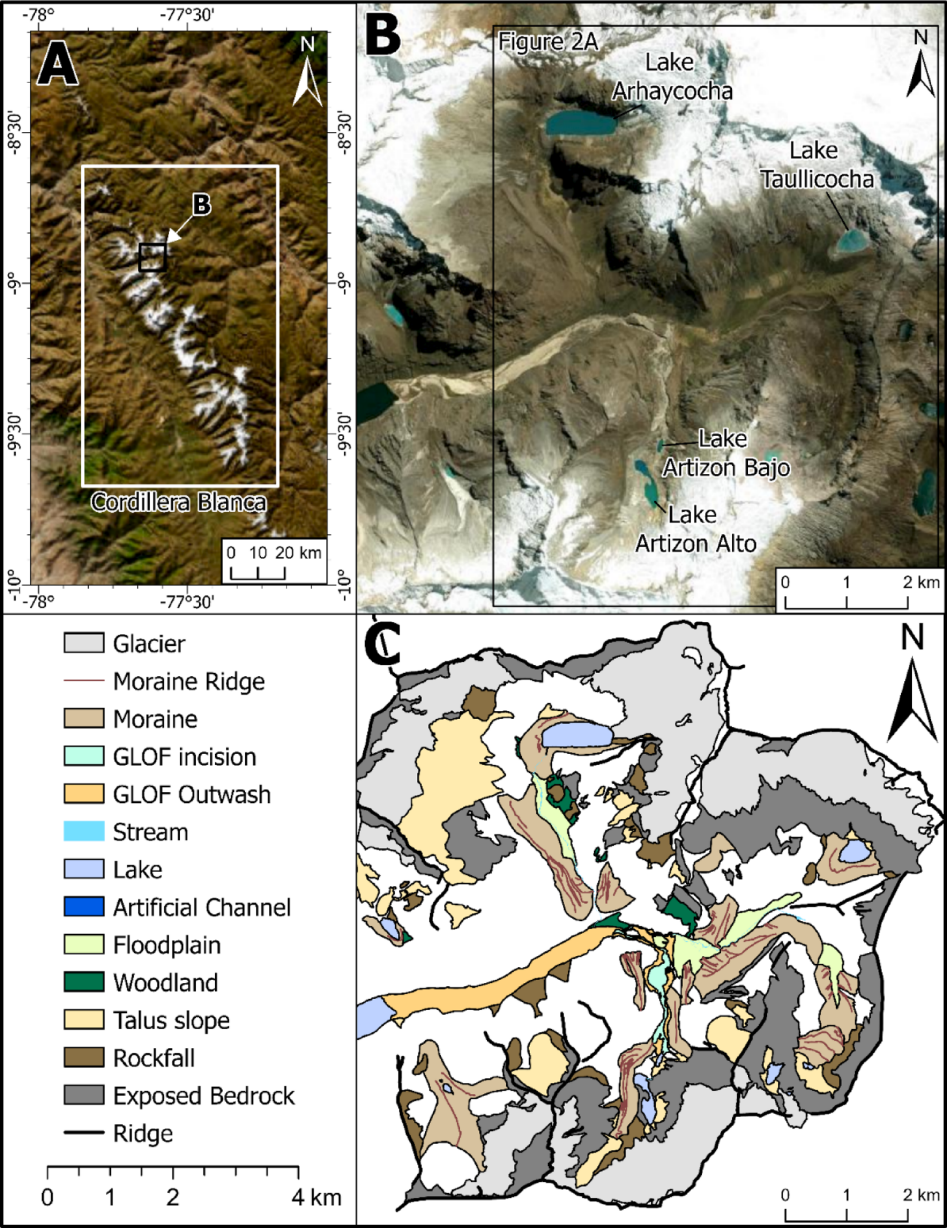
Location of the study area. **(A)** The Cordillera Blanca, Peru. **(B)** The upper Santa Cruz Valley, showing the location of the Lake Artizon, Lake Taullicocha and Lake Arhuaycocha catchments. **(C)** Overview geomorphological map of the Lake Artizon, Lake Taullicocha and Lake Arhuaycocha catchments. Figure created by the authors using ArcGIS Pro 3.5 http://pro.arcgis.com.

**Fig. 2:**
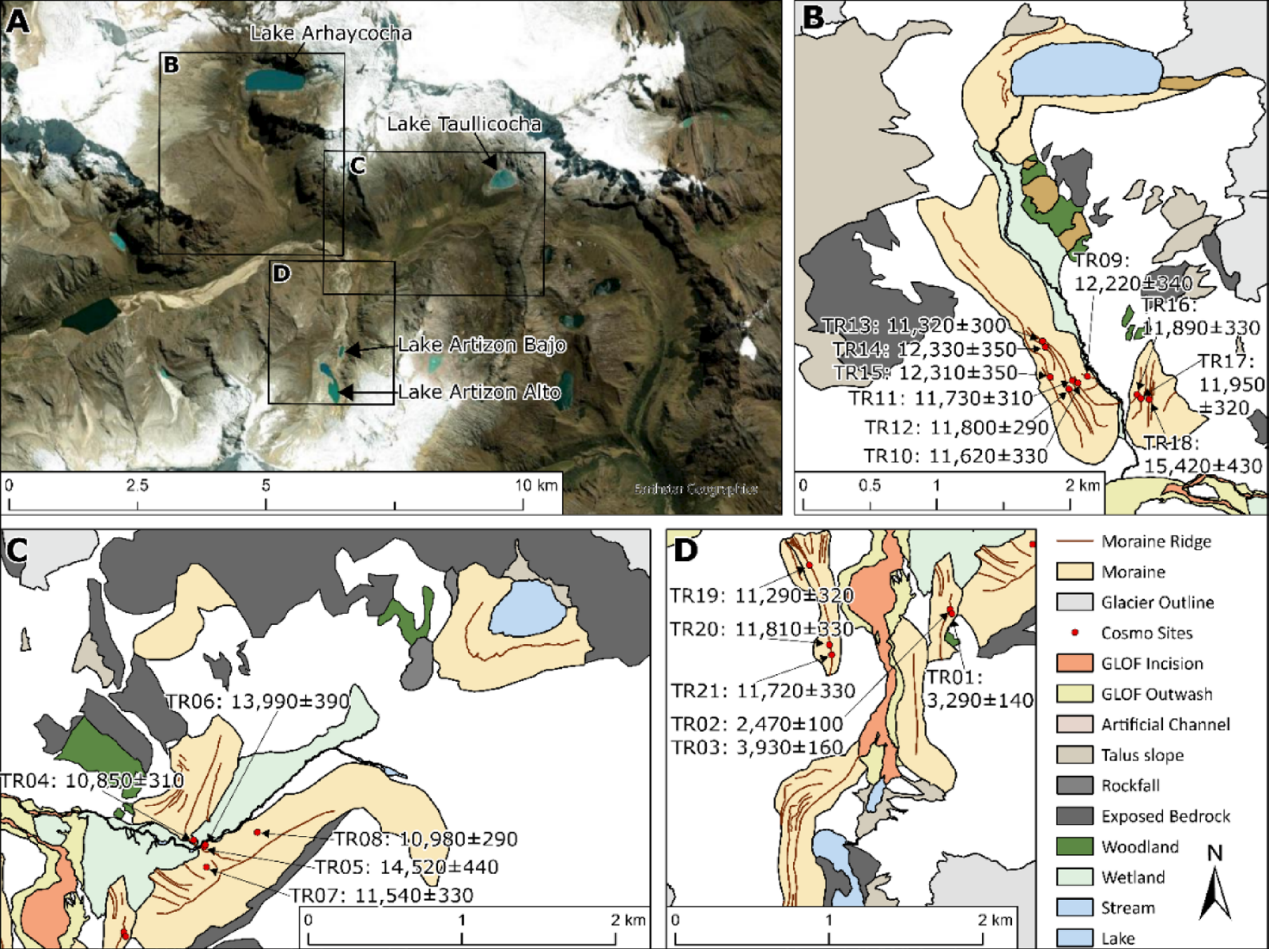
Detailed geomorphological maps showing locations of sample sites and relationship to moraine crests in the Lake Artizon, Lake Taullicocha and Lake Arhuaycocha catchments. Panel **A** shows the overall relationship within the wider catchments. Panels **B**,** C**, and **D** show detail of the moraine crests and dated samples with the three individual valleys. Location of Panel A is indicated on Fig. 1B. Figure created by the authors using ArcGIS Pro 3.5 http://pro.arcgis.com.

Here, we use ^10^Be dating on nested moraines in the Ancash region of Peru to test glacier-climate associations. We present new data from well-developed moraines in three tributary valleys in the upper Santa Cruz Valley of Peru (∼10°S at altitudes ~ 4100 to ~ 4300 m a.s.l.) (Figs. 1 and 2). We use statistical and climate reanalysis data and palaeoglacier outlines to reconstruct likely past temperature and precipitation data from the region at this time and link this to changes in the ITCZ.

## Results and discussion

### Geomorphology and cosmogenic ^10^Be measurements

Twenty-one samples were collected for ^10^Be dating from lateral and terminal moraines in three glacier catchments (Figs. 1 and 2; Figures S1 to S5; Supplementary Tables S1 to S3). These are the Lake Artizon catchment (samples TR-01, TR-02, TR-03, TR-19, TR-20, TR-21), the Lake Taullicocha catchment (samples TR-04, TR-05, TR-06, TR-07, TR-08) and the Lake Arhuaycocha catchment (samples TR-09, TR-10, TR-11, TR-12, TR-13, TR-14, TR-15, TR-16, TR-17, TR-18).

### The Lake artizon catchment

In the Lake Artizon catchment, samples TR-01, TR-02 and TR-03 were collected from the East-lateral moraine. Their ages are anomalously young in relation to all other samples at 3.3 ka, 2.5 ka and 3.9 ka, giving a mean age of 3.2 ka (Fig. 2D). Samples TR-19, TR-20 and TR-21 were collected from a sharp-crested West-lateral moraine and are statistically indistinguishable at 11.3 ka, 11.8 ka and 11.8 ka respectively, giving a moraine age of 11.6 ka (Supplementary Figure S3).

### The Lake Taullicocha catchment

In the Lake Taullicocha catchment, samples TR-04, TR-05, TR-06, TR-07 and TR-08 were collected from a series of prominent cross-valley ridges ~ 3 km down-valley of the contemporary glacier (Supplementary Figure S3). These boulders have ages of 10.8 ka, 14.5 ka, 14.0 ka, 11.5 ka and 11.0 ka respectively, giving a moraine age of 11.1 ka.

### The Lake Arhuaycocha catchment

In the Lake Arhuaycocha catchment, samples TR-09, TR-10, TR-11, TR-12, TR-13, TR-14, TR-15, TR-16, TR-17 and TR-18 were collected from prominent cross-valley moraine ridges. The outermost of these ridges, recorded by samples TR-15, TR-16, TR-17 and TR-18 has boulder ages of 12.3 ka, 11.9 ka, 11.9 ka and 15.4 ka respectively, giving a mean moraine age of 12.0 ka. Directly inside these moraines are another set of moraines, dated by samples TR-09, TR-10, TR-11, TR-12, TR-13 and TR-14, that have boulder ages of 12.2 ka, 11.6 ka, 11.7 ka, 11.8 ka, 11.3 ka and 12.3 ka respectively, giving a mean moraine age of 11.8 ka. Plotted against relative elevation, the moraine samples from the Lake Arhuaycocha catchment also record a period of rapid vertical glacier thinning between 12.0 ka and 11.8 ka (Supplementary Figure S2).

### Equilibrium line altitude (ELA) reconstruction and palaeoclimate inferences

**Fig. 3:**
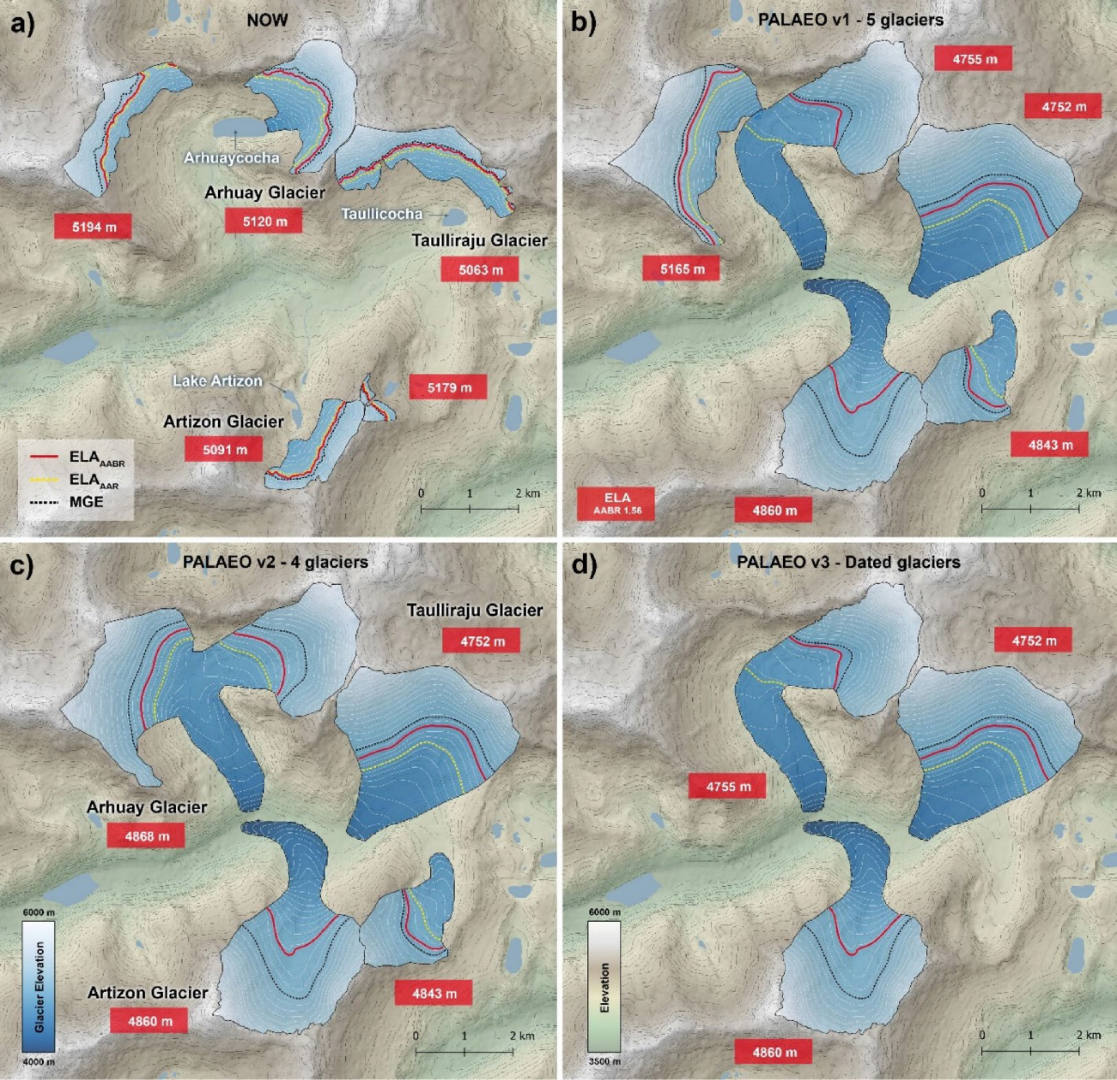
Equilibrium Line Altitude (ELA) calculated for an area-altitude balance ratio (AABR) of 1.56 and outlines of the reconstructed Palaeoglaciers. **(a)** Present-day glaciers; **(b-d)** Possible palaeo configuration with five to three glaciers. Additional ELAs calculated using accumulation area altitude and median glacier elevation (MGE) are shown for each configuration. Values for all glaciers are shown in Table S4. Figure created by the authors using ArcGIS Pro 3.5 http://pro.arcgis.com.

The ^10^Be dated moraines mark the terminal and lateral extent of the palaeoglaciers and we have considered several possible configurations for the palaeoglaciers that occupied the glacier catchments (Fig. 3a-d). We used these possible configurations to calculate Equilibrium Line Altitudes (ELAs) and palaeoclimate inferences at 11–12 ka for five-, four-, and three-glaciers configurations (Fig. 3; Supplementary Tables S4 and S5). Since our dates best constrain three of these former glacier positions, we consider the reconstructed glacier ELAs for those three glaciers (Fig. 3d). Reconstructed ELAs are between 4675 and 4835 m a.s.l. for the Arhuaycocha Glacier, between 4692 and 4832 m a.s.l. for the Taullicocha Glacier and between 4800 and 4940 m a.s.l. for the Artizon Glacier. These values represent differences of 300–400 m in elevation compared to contemporary values for the ELA, which range between 5080 and 5160 m a.s.l. for the Arhuaycocha glacier, between 5043 and 5103 m a.s.l. for the Taullicocha glacier and between 5071 and 5111 m a.s.l. for the Artizon Glacier.

### Age and timing, relationship to previous studies

The large lateral moraines in the Lake Artizon, Lake Taullicocha and Lake Arhuaycocha valleys are the furthest down-valley moraines that can be identified. Collectively, they provide moraine ages between 12.0 ka and 11.1 ka. These ages fall within the Younger Dryas Chronozone (YDC; ∼12.9 ka − 11.6 ka). Nine ^10^Be samples from the Lake Arhuaycocha catchment document a period of rapid glacier thinning and lateral contraction between 12.0 ka and 11.8 ka. Our findings show a substantial YDC glacier advance in the three valleys but no evidence of an advance during the Antarctic Cold Reversal (14.5 to 12.9 ka) in contrast to the results recorded by others^5^. Using climate model simulations these authors maintained that this substantial ACR advance was in response to reduced atmospheric CO_2_ concentrations following changes in the AMOC. They argued that northern tropical glaciers retreated during the YD in response to abrupt regional warming and reduced precipitation, while glaciers in the southern Tropical Andes, including Peru, retreated during the YD in response to warming. From this, they concluded that glacier advances during the ACR exceeded those during the YD in the region. Our ^10^Be dating contradicts those findings^5^.

Another study^13^ presented 21 ^10^Be dates from boulders on moraines in two glaciated valleys of the Cordillera Blanca, Peru. Their results indicated that the recession of glaciers after the LGM in this area of the tropical Andes was punctuated by three to four stillstands or advances during the period 12.5–7.6 ka. A recent study^14^ reported ^10^Be and ^14^C concentrations in recently exposed bedrock at the margin of four tropical glaciers in Peru. Nuclide concentrations are near zero in almost all of their samples, suggesting that these locations were never exposed during the Holocene. They concluded that many glaciers in the tropics are probably now smaller than they have been in at least 11.7 kyr, and this supports assertions about the high sensitivity of such glaciers to climate change.

In the Lake Artizon catchment, samples TR-01, TR-02 and TR-03 yielded anomalously young ages of at 3.3 ka, 2.5 ka and 3.9 ka. These samples appear to represent valley-side deposition from a debris cone that sits on top of the lateral moraine and are therefore younger than the moraine itself. Supplementary Figure S5 shows the wider geomorphological context and relationship between the debris cone and the moraine. This relationship was not apparent in the field, and since these dates are not true moraine ages they are not discussed further here.

### ELA reconstruction/paleoclimate implications

The three moraines dated here clearly formed post local LGM (after ~ 19 ka). The abundance of moraines belonging to this stage suggests that post-LGM cooling in the tropical Andes was widespread and, we argue, was coincident with the Younger Dryas Chronozone (12.8–11.5 ka)^5^. This climatic cooling event was preceded by the Antarctic Cold Reversal (ACR; 14.5–12.9 ka) in the southern hemisphere; both cooling events may be recorded by advances of Andean tropical glaciers^15^. Ice core records from Nevado Huascarán indicate periods of cooling at the onset of the ACR just prior to the start of the Younger Dryas^16^. The palaeoclimate inference from our results presented here is that there was increased precipitation during the YDC in this region and we speculate that this may reflect seasonal changes in the position of the ITCZ. This assumption is supported by proxy data and climate model simulations which indicate that there were abrupt southward shifts of the tropical rain belt during Heinrich Stadial 1 and the Younger Dryas^17^. Lake sediment and speleothem records further confirm that this southward shift during the Younger Dryas was associated with distinctly wetter conditions for both the Peruvian and Venezuelan Andes^18–20^. Climate model forcing experiments suggest that these displacements of the ITCZ during both cooling events were mainly driven by ice-sheet-induced meltwater^17^.

Our ^10^Be chronology, based on 21 boulders on moraines in three valleys of the Santa Cruz Valley, indicate a glacier advance within the YDC. Reconstructed glacier ELAs at 11–12 ka with an AABR of 1.00-2.50 are 4675 to 4835 m a.s.l. for the Arhuaycocha glacier, 4692 to 4832 m a.s.l. for the Taullicocha glacier and 4800 to 4940 m a.s.l. for the Artizon glacier. These ELAs represent an ELA depression of 300–400 m in elevation compared to contemporary values for the ELA. The paleaoclimate inference is that the glaciers in this region of the Tropical Andes advanced because of increased precipitation during the YDC and we speculate that this may represent seasonal changes in the position of the ITCZ.

The photographs in Figure S1 are all part of the field team and informed consent to the publication was obtained from team participants.

## Methods

### Data

Geomorphological mapping was conducted following well-established methods for landform mapping from remotely sensed data^21^ and field techniques^22^. The areas containing the samples sites were surveyed using a DJI Mavic 2 Pro drone in order to make detailed geomorphological maps of their spatial context and to construct topographic profiles. Detailed orthomosaics (0.2 m resolution) and Digital Elevation Models (DEMs – 0.4 m resolution) were created from the overlapping imagery collected by the drone using the Agisoft Photoscan software package which utilises an automated workflow based on Structure-from-Motion (SFM) digital photogrammetry algorithms. The horizontal and vertical accuracy of the orthomosaics and DEMs created is expected to range from 2 to 5 m based on the capabilities of the drones in-built Global Navigation Satellite System (GNSS) system^23^.

### Cosmogenic ^10^Be measurements and exposure ages

Twenty one samples were collected from granitic rocks on prominent moraine crests during fieldwork in September 2022 (Fig. 1). Samples were collected from sub-rounded and sub-angular boulders with b-axis greater than 1 m. To avoid boulders that may have been exhumed, all sampled boulders protruded more than 0.75 m above the surrounding land surface. We only sampled boulders on flat surfaces and more than 250 m away from cliff faces to ensure that sample boulders had not toppled, rotated or slid since deposition. The samples represent glacial advances in the Lake Artizon catchment, the Lake Taullicocha catchment and the Lake Arhuaycocha catchment.

We prepared samples for ^10^Be measurement in the CosmIC Laboratory, Imperial College London following established methods^24,25^. We then measured ^10^Be/^9^Be ratios at the Centre for Accelerator Science, Australian Nuclear Science and Technology Organisation (ANSTO) using published procedures^26^. We calculated exposure ages using V3 of the online calculators formerly known as the CRONUS-Earth online calculators^27^ with the Tropical Andes production rate calibration data set^28^ and the “St” scaling method^29^. Table 1 contains ^10^Be concentrations and exposure ages. The Supplemental Material contains more information on quartz and Be isolation methods, AMS measurements, and exposure age calculations. To verify accuracy and reproducibility, we also present ^10^Be concentrations for a field replicate (sample TR-01R; Table S1) and two aliquots of the laboratory intercomparison material CRONUS-A (Jull et al., 2015; Table S1). The “St” scaling scheme was used as it is the most appropriate for this area of the Earth.

It is difficult to assess the influence of snow cover on the moraine ages because there is limited assessment of current or past snow cover and thickness from the Peruvian Andes. However, recent research^30^ has shown that snow dynamics in the Cordillera Blanca are characterised by a highly ephemeral snowpack at elevations below ~ 5000 m a.s.l. Here, the snowpack is thin and melts in hours to days, with the snowpack duration and thickness increasing with elevation. All our moraine sampling sites are below 5000 m elevation and we therefore assess the impact of long-duration snow cover on moraine ages as being minimal.

### Equilibrium line altitude (ELA) reconstruction and palaeoclimate inferences

The former surface and Equilibrium Line Altitude (ELA) of the palaeo-glaciers with available moraine dating were calculated using the Palaeoice 2.0 toolbox in ArcGIS Pro^31^, which is a revised version of another tool^32,33^. This approach, based on modern-day glacier thickness data^34^ and a 10-meter-resolution digital surface model (DSM) from NEXTMap^®^, involves several steps: (i) Modern-day ice thickness data were subtracted to the DSM, recovering the former subglacial bed topography. (ii) The palaeo-glacier outlines were then manually drawn, based on geomorphological evidence such as terminal and lateral moraines. (iii) The digitised outlines and glacier flowlines were used to retrieve the ice thickness points and reconstruct the palaeo-glaciers surface. This was done using *Palaeoice 2.0* with an ice thickness point resolution along flowlines of 50 m and a shear stress of 100 KPa. (iv) Lastly, ice surface DSMs and glaciers outlines were used to recover the ELAs of the palaeo-glaciers, with an area–altitude balance ratio (AABR) approach^35^. We used the proposed AABR value of 1.56^36^ and, to incorporate the uncertainty in estimating the balance ratio of tropical glaciers lacking mass balance, we calculated ELA also with a broader range of AABR 1 to 2.5 previously applied to Northern Perù^4^. This range encompasses potential ELA values that could fall within these limits to give an insight on uncertainties. Additional results from the accumulation area ratio AAR method and median glacier elevation (MGE) have been calculated using the same tool (Fig. 3 and Supplementary Table S3).

Palaeoclimate inferences were drawn from the palaeo-ELA using the ArcGIS tool^31^. Given the proximity of the glaciers to each other in the study area, we utilised average ELA values. Summer temperature and annual precipitation at the ELA are related by a polynomial equation^37^. Thus, by knowing two of the three variables it is possible to calculate the third one. This method has been already successfully applied elsewhere^38,39^, the latter within a palaeoclimatic framework^39^. We retrieved temperatures from the closest cell of the ERA5 reanalysis datasets to the study area and calculated the 1961–1990 summer mean. These temperatures were corrected to take into account the elevation difference between ERA5 grid cell (3895 m) and the present-day ELA (5129 m). This was done using an environmental lapse rate of 0.45 °C/100m (Eq. 1), typical for austral summer in the studied region^40^. Then, we reconstructed precipitation at the present-day ELA (P_PD_ELA_) using present-day ELA and the altitude-corrected ERA5 mean summer temperatures (T_PD_ELA_summer_; Eq. 2).

1 $$\:\text{T}_{\text{PD}\_\text{E}\text{L}\text{A}\_\text{s}\text{u}\text{m}\text{m}\text{e}\text{r}}\:=\:\text{T}_{\_\text{E}\text{R}\text{A}5}\:-\:\left(\right(\text{E}\text{L}\text{A}_{\text{P}\text{D}}\: -\:\text{H}_{\text{E}\text{R}\text{A}5}\left)\:\text{*}\:\right(0.45/100\left)\right)$$

2 $$\:\text{P}_{\text{P}\text{D}\_\text{E}\text{L}\text{A}}\:=\:5.87\:\cdot\:\text{T}_{\text{P}\text{D}\_\text{E}\text{L}\text{A}\_\text{s}\text{u}\text{m}\text{m}\text{e}\text{r}}\,^2+\:230\:\cdot\:\text{T}_{\text{P}\text{D}\_\text{E}\text{L}\text{A}\_\text{s}\text{u}\text{m}\text{m}\text{e}\text{r}}\:+\:966$$

## Result at PD ELA (5129 m)

- T_PD_ELA_summer_ = 1.18 °C.
- P_PD_ELA_ = 1244.8 mm yr^−1^.

The final step was a calculation of the temperature difference between palaeo- and present-day temperatures, taking into account the elevation difference between palaeo- and present-day ELA and hypothesising different total annual precipitation amounts. Present-day precipitation (P_PD_ELA_) was increased/decreased at 5% steps (Supplementary Table S4) and used in Eq. 2 to calculate the respective temperatures. All calculations were performed with three ELA values, corresponding to the three different glacier configurations (see Supplementary Table S4).

## Supplementary Information

Below is the link to the electronic supplementary material.

Supplementary Material 1

## Data Availability

The datasets used and/or analysed during the current study available from the corresponding author on reasonable request.
