# Supplementary material for: Younger Dryas glacier advances in the tropical Andes driven by increased precipitation
Source: Sci Rep. 2025 Aug 22;15:30832. doi: 10.1038/s41598-025-16603-3 (PMC12370917; doi:10.1038/s41598-025-16603-3)
Supplement: Supplementary file 1 — Supplementary Material 1 [file 41598_2025_16603_MOESM1_ESM.pdf]

Glasser et al., *Scientific Reports*

List of Supplementary Figures

Figure S1. Photographs of samples collected for cosmogenic isotope dating from moraine crests in the Lake Artizon, Lake Taullicocha and Lake Arhaycocha catchments.

Figure S2. Moraine ridges in cross-section, annotated with moraine ages in the three catchments with sample locations and dates shown. Topographic profiles were extracted from DEMs constructed from drone survey of the area in September 2022. See Figure 3 for locations).

Figure S3. View towards Taullicocha Glacier. The prominent cross-valley moraines are dated by samples TR-04, TR-05 and TR-06 (ages of 10.9 ka 14.5 ka and 14.0 ka respectively). Lake Taullicocha is not visible because it is hidden by the large moraines in the background.

Figure S4, Probability density functions of dates obtained from moraine crests in the Lake Artizon, Lake Taullicocha and Lake Arhaycocha catchments.

Figure S5 Wider geomorphological context for samples TR-01 to TR-03, showing the relationship between a valley-side debris cone and the moraine. Figure created by the authors using Google Earth. Earth Versions – Google Earth

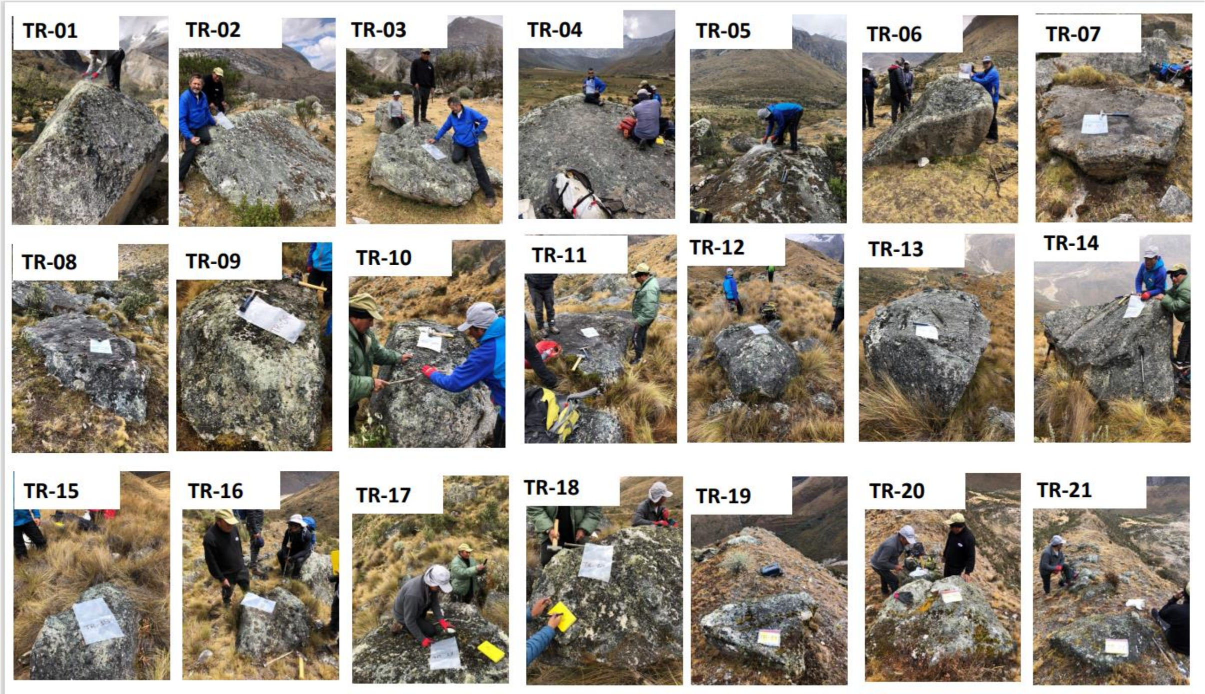

Figure S1. Photographs of samples collected for cosmogenic isotope dating from moraine crests in the Lake Artizon, Lake Taullicocha and Lake Arhaycocha catchments. These photographs are all part of the field team and informed consent to the publication was obtained from team participants.

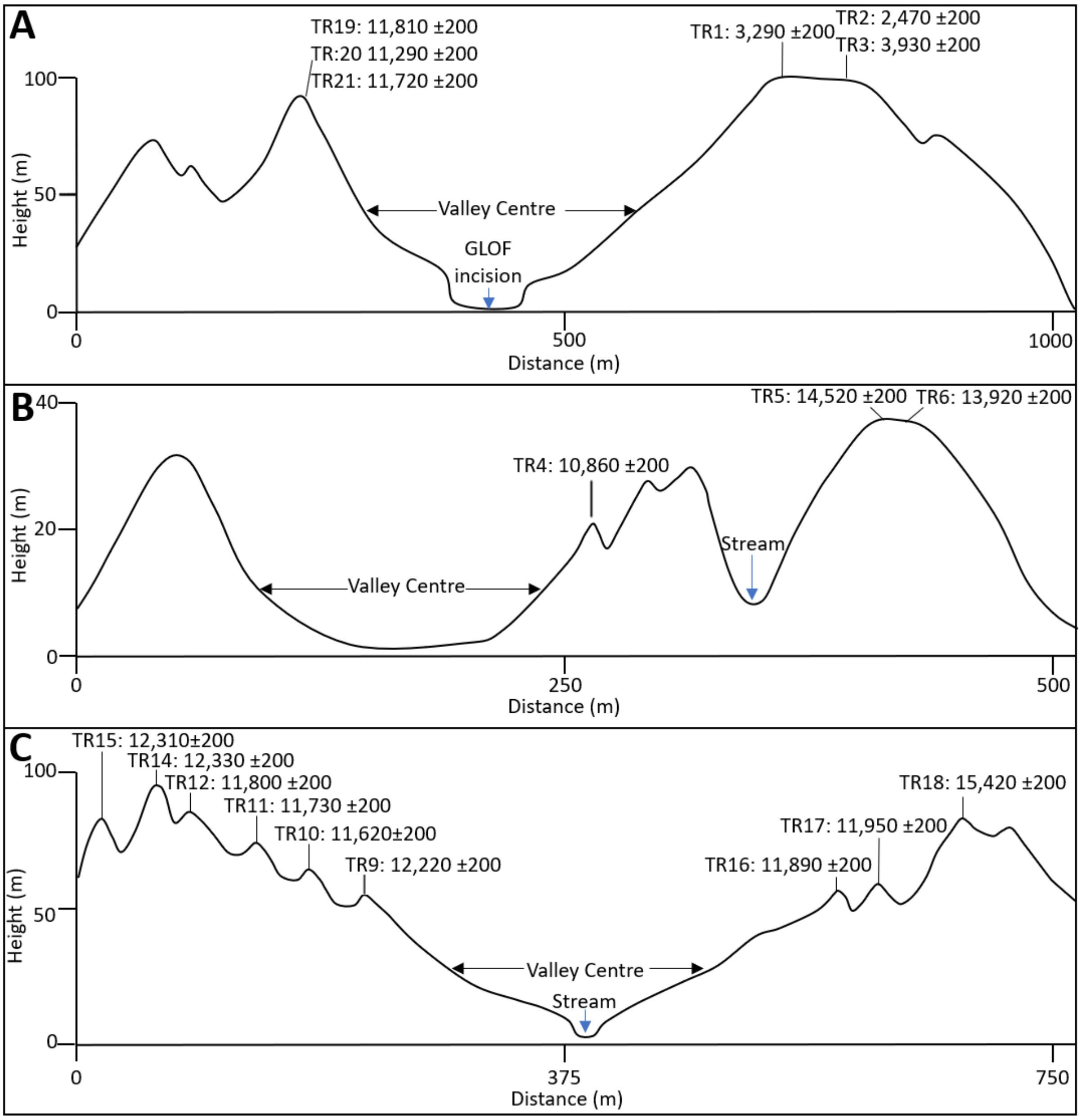

Figure S2. Moraine ridges in cross-section, annotated with moraine ages. The topographic profiles were extracted from DEMs constructed from a drone survey of the area in September 2022. See Figure 3 for locations. Note vertical exaggeration.

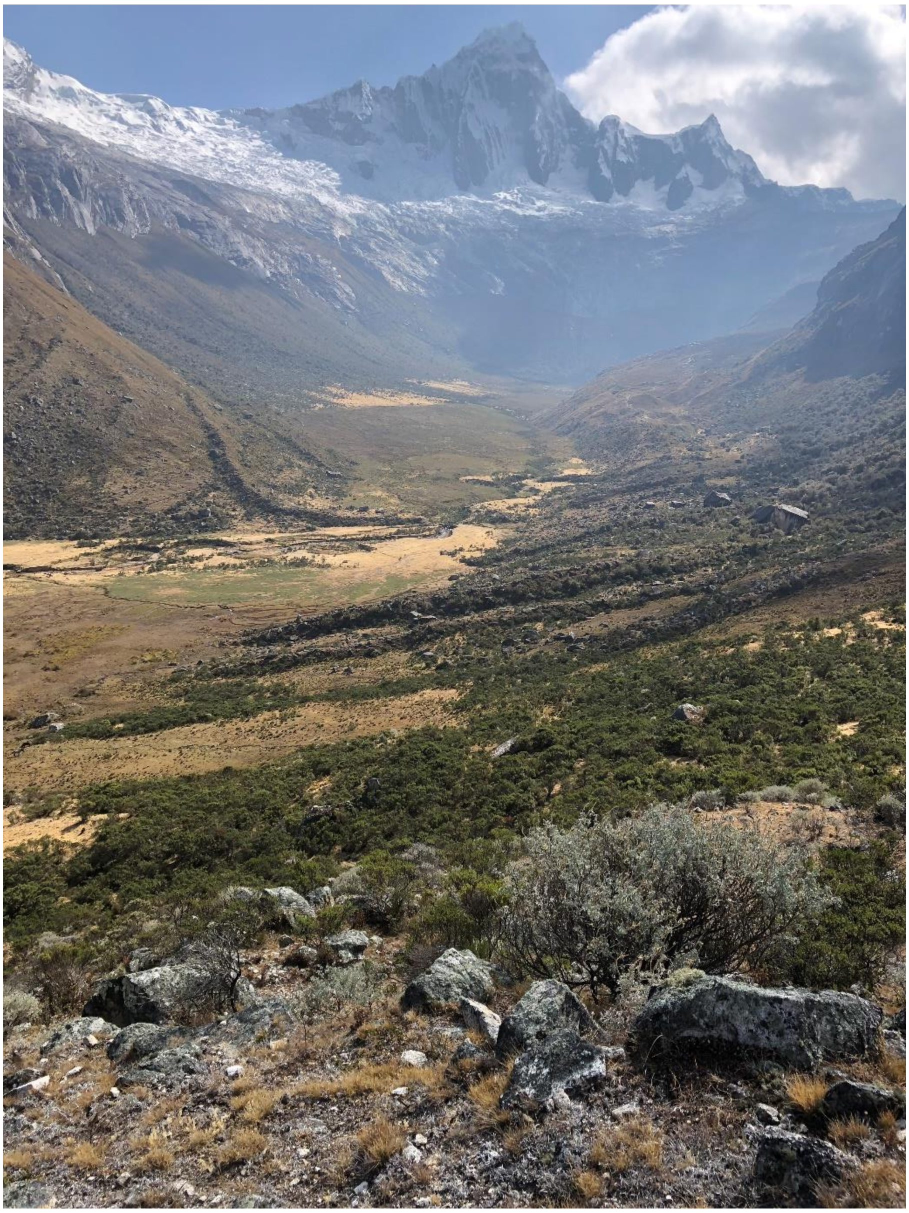

Figure S3. View towards Taullicocha Glacier. The prominent cross-valley moraines are dated by samples TR-04, TR-05 and TR-06 (ages of 10.9 ka 14.5 ka and 14.0 ka respectively). Lake Taullicocha is not visible because it is hidden by the large moraines in the background.

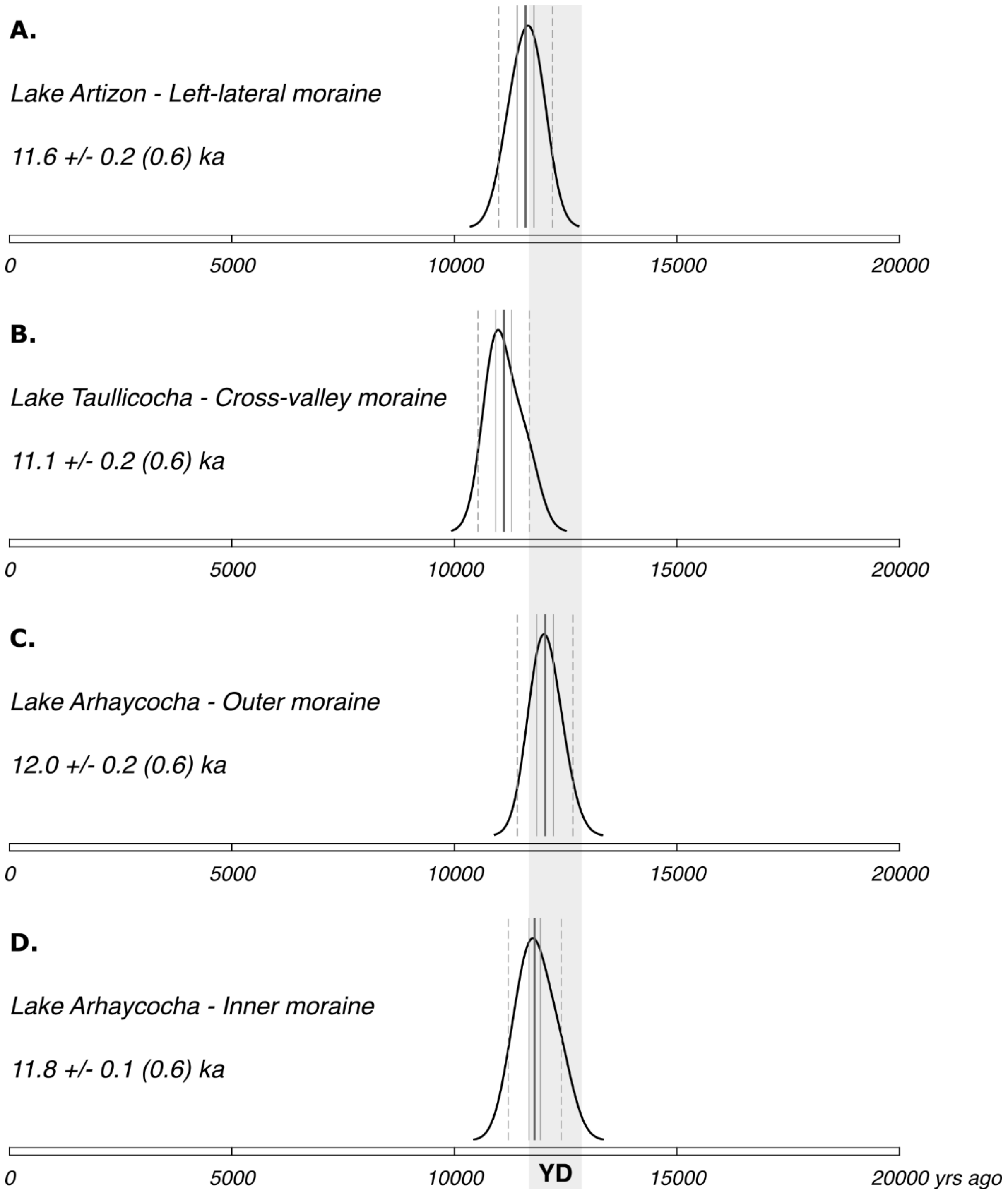

Figure S4, Probability density functions of dates obtained from moraine crests in the Lake Artizon, Lake Taullicocha and Lake Arhaycocha catchments. The values in brackets, e.g., “(0.6)”, are the external uncertainties that include the propagated production rate uncertainty. Summary statistics are included in Tables S1 to S3.

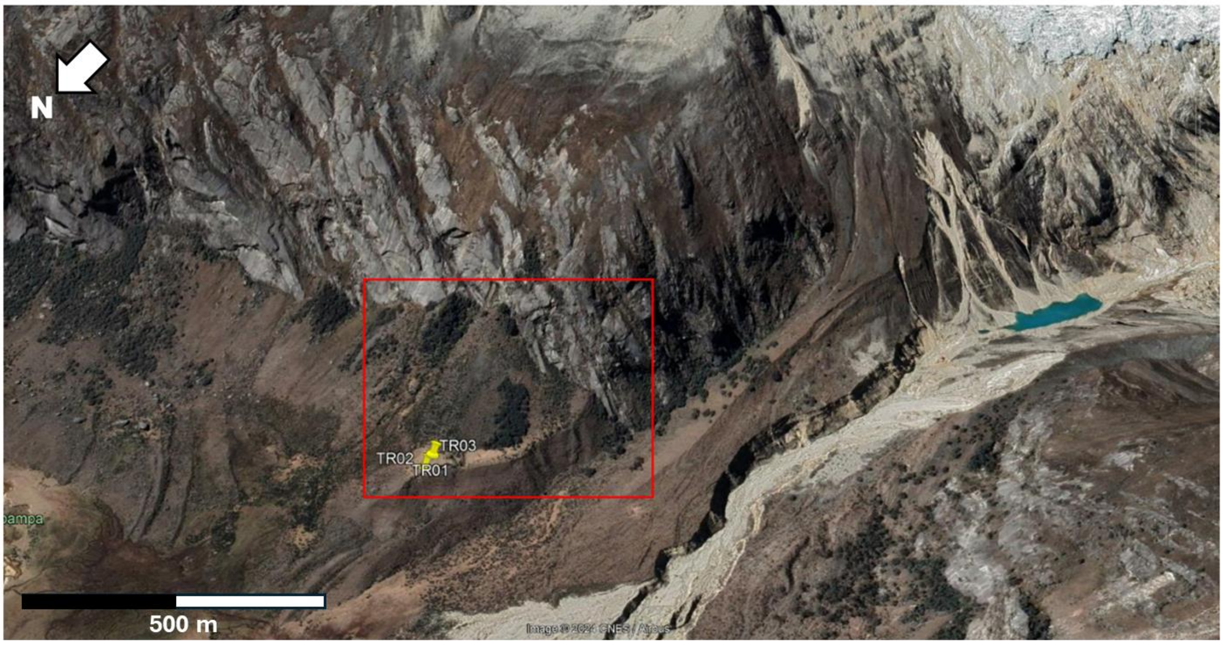

Figure S5 Wider geomorphological context for samples TR-01 to TR-03, showing the relationship between a valley-side debris cone and the moraine. Area in red box expanded below. Figure created by the authors using Google Earth. Earth Versions – Google Earth

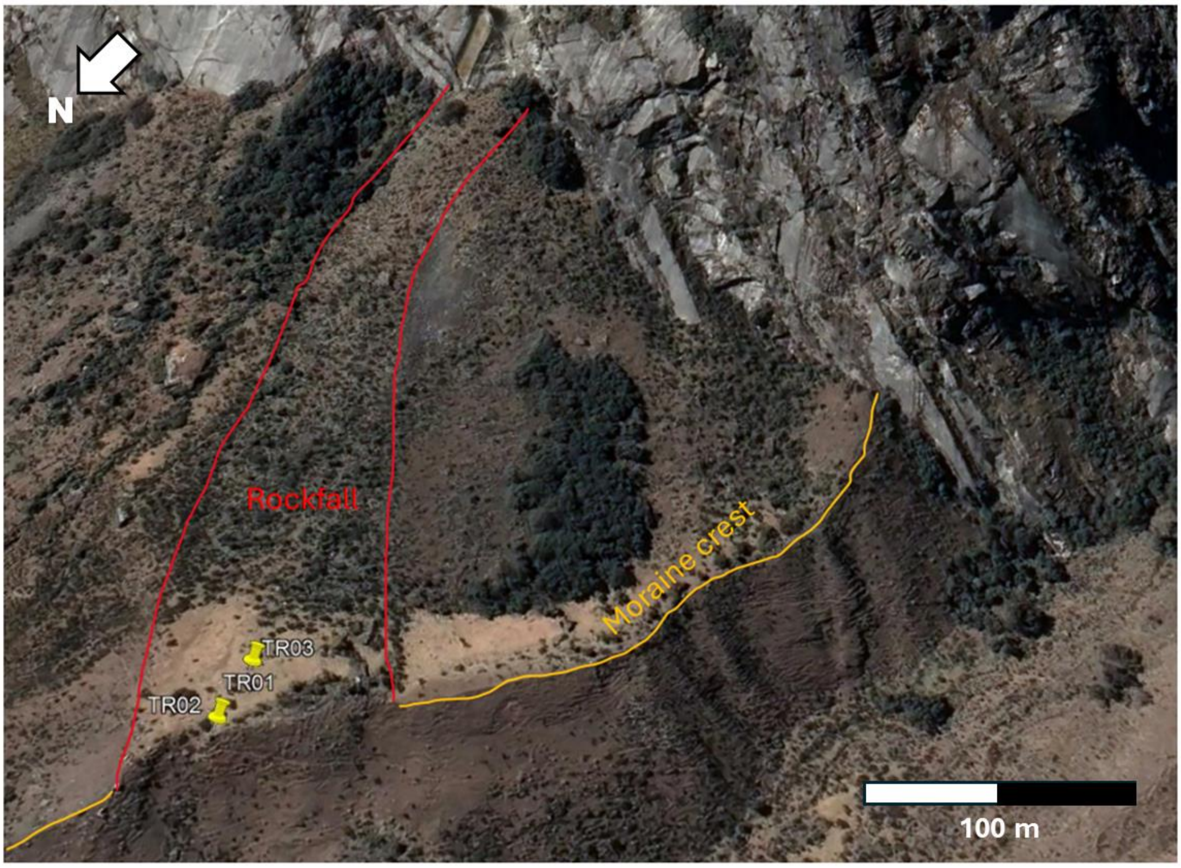

Figure S5 (continued) Wider geomorphological context for samples TR-01 to TR-03, showing the relationship between a valley-side debris cone and the moraine. Figure created by the authors using Google Earth. [Earth Versions – Google Earth]

List of Supplementary Tables

Table S1. Sample information (location, elevation, topographic shielding factor, and sample thickness), ^{10}Be concentrations, and ^{10}Be ages. Exposure ages calculated with the "St" (Stone, 2000) scaling method and Tropical Andes production rate calibration dataset (Kelly et al., 2015). TR01R is a replicate of TR01 and the average and standard deviation of both ^{10}Be concentrations is used to calculate the exposure age for this sample. See Supplementary Material for more information on laboratory methods and exposure age calculations.

Table S2: Beryllium analytical data (inputs for ^{10}Be concentration calculations). $^{10}Be/^{9}Be$ ratios normalised to the primary standard KN-5-3 with a nominal value of 6.320 x 10-12 (Nishiizumi et al., 2007). ^{10}Be concentrations corrected for background concentrations -using the average number of ^{10}Be atoms of six process blanks (sample IDs with BLK prefix; 27409 ± 11906 (1 sigma), with uncertainties propagated in quadrature). ~~Sample~~ TR-12 Sample contained native Be, so the total amount of Be was quantified with ICP-OES analysis of a dissolution solution aliquot. TR01R is

a replicate of TR01. Sample IDs with STDA prefix are aliquots of the CRONUS-A interlaboratory comparison material with a consensus ^{10}Be concentration of 3.42 ± 0.1 x 10^7 atoms g^{-1} (Jull et al., 2015). ICL22-1A and -1B are directly-precipitated carrier blanks (ICL22-1).

Table S3. Summary statistics for ^{10}Be dates reported in this paper.

Table S4. Equilibrium Line Altitudes (m a.s.l.) of the Arhuay ~~G~~laciers, Taulliraju gGlacier, Artizon gGlacier and the additional unnamed glacier in the study area and reconstructed under different palaeo-configurations and ELA methods: area-altitude balance ratio (AABR) with a global value of 1.56 (Oien et al., 2022) and a range of values for tropical glaciers (Lee et al., 2022), accumulation area ratio (AAR) of 0.67 and Median Glacier Elevation (MGE). RGI-ID (RGI Consortium, 2023) of each glacier is shown on the table. *Reconstruction that includes the scenario where Arhuay ~~G~~lacier and Arhuay East ~~g~~Glacier were united into a single glacial body (Fig. 3.c).

Table S5: Temperature difference between present-day and the Early-Holocene, according to the five-, four-, and three-glaciers configurations and for different precipitation amounts. Temperature differences show a temperature rise spanning from 0.15 °C to 2.58 °C from Early-Holocene to present-day.

Sample ID	Latitude	Longitude	Elevation (m)	Thickness (cm)	Shielding	[^{10}Be] (at/g)*	1σ Uncert. [^{10}Be] (at/g)	Age (a)**	1σ Int. Uncert. Age (a) **	1σ Ext. Uncert. Age (a) **
TR01						101251	2992			
TR01R***	-8.9241	-77.6108	4302	2.3	0.958	95376	3583			
TR01 Average						98313	4154	3289	139	213
TR02	-8.9239	-77.6109	4298	3.9	0.958	72811	3046	2472	103	160
TR03	-8.9241	-77.6108	4302	3.1	0.958	116813	4615	3934	156	248
TR04	-8.9185	-77.6067	4171	2.8	0.974	308749	8778	10855	309	618
TR05	-8.9189	-77.6061	4176	2.0	0.963	411726	12535	14523	444	843
TR06	-8.9188	-77.6060	4169	3.5	0.963	390575	10907	13992	392	794
TR07	-8.9201	-77.6059	4190	2.1	0.949	324569	9165	11546	327	657
TR08	-8.9181	-77.6029	4210	2.9	0.952	310507	8167	10984	290	614
TR09	-8.9087	-77.6270	4208	2.5	0.964	350760	9809	12220	343	693
TR10	-8.9091	-77.6276	4246	1.4	0.978	347594	9724	11621	326	659
TR11	-8.9090	-77.6280	4260	1.9	0.965	346832	9199	11732	312	657
TR12	-8.9096	-77.6282	4282	2.0	0.975	355502	8691	11797	289	650
TR13	-8.9063	-77.6300	4367	2.1	0.981	356606	9398	11319	299	633
TR14	-8.9067	-77.6298	4354	4.5	0.987	380315	10629	12326	346	699
TR15	-8.9087	-77.6295	4290	2.3	0.973	370557	10359	12305	345	698
TR16	-8.9100	-77.6236	4200	2.2	0.963	340351	9526	11888	334	675
TR17	-8.9102	-77.6233	4216	2.7	0.960	342222	8988	11951	315	668
TR18	-8.9103	-77.6228	4229	2.5	0.953	441655	12322	15420	432	875
TR19	-8.9212	-77.6192	4277	2.1	0.985	342993	9593	11294	317	641
TR20	-8.9259	-77.6014	4320	1.6	0.954	355438	9945	11808	331	670
TR21	-8.9265	-77.6179	4355	2.0	0.932	349434	9831	11722	331	666

* Normalized to ^{10}Be AMS standard KN-5-3 with nominal value of 6.320e-12

** Age calculations using standard atmosphere, density 2.7 g cm^{-3}, zero erosion rate, Tropical Andes production rate calibration dataset for Huancane IIa moraines (Kelly et al., 2015; site HUANCANE2A in ICE-D database), and St scaling scheme with the CRONUS online calculator v3 (Balco et al., 2008; wrapper: 3.0.2; get_age: 3.0.2; muons: 1A, alpha = 1; validate: validate_v3_input.m - 3.0; consts: 2020-08-26)

*** TR01R is a replicate of TR01; the average and standard deviation of both [^{10}Be] is used for age calculation

Table S1

Chemistry ID	AMS ID	Quartz Mass (g)	Carrier Solution Mass (g)	Carrier Solution Concentration (μg Be/g)	Carrier Mass (μg Be)	^{9}Be (atoms)	Measured ^{10}Be/^{9}Be*	1σ Measured ^{10}Be/^{9}Be*	Blank Correction ID	Total ^{10}Be in Sample (atoms)	1σ Total ^{10}Be in Sample (atoms)	Background-corrected Total ^{10}Be in Sample (atoms) **	1σ Background corrected Total ^{10}Be in sample	[^{10}Be] (atoms/g)	1σ [^{10}Be] (atoms/g)
TR-01	XBE1854	25.492	1.4826	178	264	1.76E+19	1.48E-13	4.27E-15	Blank average	2608498	75332	2581089	76267	101251	2992
TR-02R	XBE1855	10.658	1.4626	178	260	1.74E+19	4.62E-14	1.74E-15	Blank average	803429	30203	776020	32465	72811	3046
TR-03R	XBE1856	9.933	1.4676	178	261	1.75E+19	6.80E-14	2.54E-15	Blank average	1187713	44267	1160305	45840	116813	4615
TR-04R	XBE1857	10.323	1.4643	178	261	1.74E+19	1.85E-13	5.16E-15	Blank average	3214628	89834	3187220	90620	308749	8778
TR-05R	XBE1858	5.131	1.4687	178	261	1.75E+19	1.23E-13	3.62E-15	Blank average	2139975	63206	2112567	64318	411726	12535
TR-06R	XBE1859	7.096	1.4699	178	262	1.75E+19	1.60E-13	4.37E-15	Blank average	2798927	76473	2771518	77394	390575	10907
TR-07	XBE1860	15.059	1.4677	178	261	1.75E+19	2.82E-13	7.88E-15	Blank average	4915100	137496	4887692	138010	324569	9165
TR-08	XBE1861	15.124	1.4686	178	261	1.75E+19	2.70E-13	7.04E-15	Blank average	4723521	122938	4696112	123513	310507	8167
TR-09	XBE1862	15.102	1.4673	178	261	1.75E+19	3.05E-13	8.46E-15	Blank average	5324594	147656	5297185	148135	350760	9809
TR-10	XBE1863	15.189	1.4650	178	261	1.74E+19	3.05E-13	8.45E-15	Blank average	5307012	147220	5279603	147701	347594	9724
TR-11	XBE1864	15.004	1.4744	178	262	1.75E+19	2.98E-13	7.84E-15	Blank average	5231282	137509	5203874	138024	346832	9199
TR-12R***	XBE1865	14.181	1.4708	178	329	2.20E+19	2.31E-13	5.58E-15	Blank average	5068780	122671	5041372	123247	355502	8691
TR-13	XBE1867	15.052	1.4634	178	260	1.74E+19	3.10E-13	8.10E-15	Blank average	5395035	140950	5367626	141452	356606	9398
TR-14	XBE1868	15.038	1.4681	178	261	1.75E+19	3.29E-13	9.13E-15	Blank average	5746586	159400	5719177	159844	380315	10629
TR-15	XBE1869	15.091	1.4687	178	261	1.75E+19	3.22E-13	8.92E-15	Blank average	5619488	155875	5592079	156329	370557	10359
TR-16	XBE1870	15.025	1.4701	178	262	1.75E+19	2.94E-13	8.16E-15	Blank average	5141186	142633	5113778	143129	340351	9526
TR-17	XBE1871	15.057	1.4668	178	261	1.74E+19	2.97E-13	7.73E-15	Blank average	5180241	134802	5152832	135327	342222	8988
TR-18	XBE1872	15.084	1.4729	178	262	1.75E+19	3.82E-13	1.06E-14	Blank average	6689334	185485	6661925	185867	441655	12322
TR-19	XBE1873	15.032	1.4686	178	261	1.75E+19	2.97E-13	8.23E-15	Blank average	5183279	143712	5155870	144204	342993	9593
TR-20	XBE1874	15.079	1.4663	178	261	1.74E+19	3.09E-13	8.57E-15	Blank average	5387065	149494	5359656	149968	355438	9945
TR-21	XBE1875	11.823	1.4681	178	261	1.75E+19	2.38E-13	6.62E-15	Blank average	4158762	115615	4131353	116226	349434	9831
TR-01R****	XBE1876	11.011	1.4709	178	262	1.75E+19	6.16E-14	2.15E-15	Blank average	1077591	37613	1050183	39452	95376	3583
STDA050923*****	XBE1853	0.506	1.4682	178	261	1.75E+19	1.01E-12	2.51E-14	Blank average	17588974	438193	17564584	438194	34712616	865997
STDA240723*****	XBE1877	0.559	1.4648	178	261	1.74E+19	1.13E-12	3.13E-14	Blank average	19736546	545305	19709137	545435	35257848	975733
BLK290823A	XBE1836	0	1.4660	178	261	1.74E+19	1.46E-15	3.79E-16		25516	6608				
BLK290823B	XBE1844	0	1.4696	178	262	1.75E+19	1.33E-15	2.63E-16		23325	4598				
BLK050923A	XBE1845	0	1.4679	178	261	1.75E+19	1.33E-15	2.68E-16		23281	4679				
BLK050923B	XBE1851	0	1.4651	178	261	1.74E+19	1.46E-15	2.25E-16		25439	3913				
BLK010823	XBE1852	0	1.4611	178	260	1.74E+19	2.92E-15	4.44E-16		50689	7709				
BLK240723	XBE1866	0	1.4621	178	260	1.74E+19	9.32E-16	2.27E-16		16202	3943				
Blank average										27409	11906				
ICL22-1A******	XBE1878	0	1.4696	178	262	1.75E+19	2.22E-16	1.28E-16		3880	2241				
ICL22-1B******	XBE1879	0	1.4700	178	262	1.75E+19	2.55E-16	1.14E-16		4461	1997				

* Normalized to ^{10}Be AMS standard KN-5-3 with nominal value of 6.320e-12

** Corrected for background using average of 6 process blanks (sample IDs with BLK prefix) 27409 +/- 11906 (1σ) 10Be atoms, with uncertainties propogated in quadrature

*** Sample contained native Be; total Be quantified with ICP-OES analysis of dissolution solution aliquot

**** TR01R is a replicate of TR01

***** CRONUS-A reference material with [10Be] = 3.42 ± 0.1 x 10^7 atoms/g (Jull et al., 2015)

****** Directly-precipitated carrier blank (ICL22-1)

Table S2

[illegible]

Catchment	Landform	Sample	Sample Age (yr)*	Int. error (yr)*	Ext. error (yr)*	Moraine Age (yr)	Int. error (yr)	Ext. error (yr)	N	Reduced chi-squared	p-value	Probability Younger Dryas	Probability Antarctic Cold Reversal
Lake Artizon													
	Right-lateral moraine												
		TR01	3289	139	213	3232	733	-	3	37.42	0.0000	-	-
		TR02	2472	103	160								
		TR03	3934	156	248								
	Left-lateral moraine												
		TR19	11294	317	641	11602	188	602	3	0.73	0.3480	**0.42**	0.01
		TR20	11808	331	670								
		TR21	11722	331	666								
Lake Taullicocha													
	Cross-valley moraine												
		TR04	10855	309	618	11124	178	577	3	1.33	0.3256	**0.16**	0.00
		TR05	*14523*	*444*	*843*								
		TR06	*13992*	*392*	*794*								
		TR07	11546	327	657								
		TR08	10984	290	614								
Lake Arhaycocha													
	Outer moraine												
		TR15	12305	345	698	12059	191	625	3	0.44	0.6171	**0.63**	0.07
		TR16	11888	334	675								
		TR17	11951	315	668								
		TR18	*15420*	*432*	*875*								
	Inner moraine												
		TR09	12220	343	693	11825	130	597	6	1.36	0.2519	**0.55**	0.02
		TR10	11621	326	659								
		TR11	11732	312	657								
		TR12	11797	289	650								
		TR13	11319	299	633								
		TR14	12326	346	699								

* Local production rate calibration (Kelly et al., 2015) and St scaling scheme

Italics = outliers

Table S3

**Present ELA (m a.s.l)**	**AABR** 1.56	**AABR Range** 1.00 - 2.50	**AAR** 1.67	**MGE**	**RGI ID**
Arhuay East Glacier	5194	5174 - 5234	5174	5254	RGI2000-v7.0-G-16-00169
Arhuay Glacier	5120	5080 - 5160	5060	5160	RGI2000-v7.0-G-16-01434
Taulliraju Glacier	5063	5043 - 5103	5023	5083	RGI2000-v7.0-G-16-01435, RGI2000-v7.0-G-16-01440
Artizon Glacier	5091	5071 - 5111	5071	5131	RGI2000-v7.0-G-16-00232
Unnamed Glacier	5179	5159 - 5199	5159	5199	RGI2000-v7.0-G-16-00229
**Palaeo ELA**	**AABR** 1.56	**AABR Range** 1.00 - 2.50	**AAR** 1.67	**MGE**	**RGI ID**
Arhuay East Glacier	5165	5070 - 5170	5050	5170	RGI2000-v7.0-G-16-00169
Arhuay Glacier	4755	4675 - 4835	4535	4795	RGI2000-v7.0-G-16-01434
Arhuay Glacier*	4868	4808 - 4948	4728	4988	N.A.
Taulliraju Glacier	4752	4692 - 4832	4592	4832	RGI2000-v7.0-G-16-01435, RGI2000-v7.0-G-16-01440
Artizon Glacier	4860	4800 - 4940	4860	5000	RGI2000-v7.0-G-16-00232
Unnamed Glacier	4843	4803 - 4883	4763	4883	RGI2000-v7.0-G-16-00229

Table S4

	5 glaciers	4 glaciers	3 glaciers
	ELA = 4875	ELA = 4830	ELA = 4789
	**Delta T PD-YD [° C]**	**Delta T PD-YD [° C]**	**Delta T PD-YD [° C]**
**T PR +20%**	0.15	0.35	0.53
**T PR +15%**	0.39	0.60	0.78
**T PR +10%**	0.64	0.84	1.03
**T PR +5%**	0.89	1.09	1.28
**T PR 0%**	1.14	1.35	1.53
**T PR -5%**	1.40	1.60	1.79
**T PR -10%**	1.66	1.86	2.05
**T PR -15%**	1.93	2.13	2.31
**T PR -20%**	2.19	2.39	2.58

Table S5

Supplementary File 1: Processing steps for cosmogenic nuclide dating

Quartz and Be isolation, AMS measurements

We prepared purified quartz samples for cosmogenic nuclide analysis in the CosmIC Laboratory, Imperial College London. We applied typical quartz isolation procedures (e.g. Kohl and Nishiizumi, 1992), including crushing, milling, sieving, magnetic separation, and HCl leaching. Quartz purity was tested using inductively coupled plasma optical emission spectrometry (ICP-OES). We performed isotope dilution chemistry for ^{10}Be analysis using methods similar to Corbett et al. (2016). For each sample, we dissolved between 5.1 and 25.4 g of pure quartz (Table S1) and approximately 1450 mg of low-background ^{9}Be carrier (178 µg Be g^{-1}; $^{10}Be/^{9}Be = 2.39 \times 10^{-16} \pm 2.35 \times 10^{-17}$; Table S1) using concentrated HF and HNO_3. Six procedural blanks (carrier-only) and two aliquots of ^{9}Be carrier blanks (carrier precipitated prior to sample drydown and ignition without any column chemistry) were prepared alongside the samples (Table S1). To verify accuracy and reproducibility, we also prepared two aliquots (~0.5 g of quartz) of the laboratory intercomparison material CRONUS-A (Jull et al., 2015) and a field replicate (sample TR-01R; Table S1).

We measured $^{10}Be/^{9}Be$ ratios at the Centre for Accelerator Science, Australian Nuclear Science and Technology Organisation (ANSTO) using procedures described in Wilcken et al. (2022). The $^{10}Be/^{9}Be$ measurements are normalised to the KN-5-3 standard with an assumed ratio of 6.320×10^{-12} (t1/2=1.36 Ma, Nishiizumi et al., 2007; Table 1).

Blank correction and reproducibility

^{10}Be concentrations were corrected using the mean and standard deviation of the total number of ^{10}Be atoms measured for the six procedural blanks (27409 ± 11906 (1σ)), with uncertainties propagated in quadrature. The two measurements of TR-01 vary by 6.1 %, therefore we use the average and standard deviation of the two ^{10}Be concentrations (Table 1) in the exposure age calculations described below. The two aliquots of CRONUS-A yield ^{10}Be concentrations of 3.47×10^{7} ($\pm 8.66 \times 10^{5}$) and 3.53×10^{7} ($\pm 9.76 \times 10^{5}$) atoms g^{-1} which are ~1.5 and 3% different than, and therefore consistent with, the consensus value of $3.42 \pm 0.1 \times 10^{7}$ atoms g^{-1} of Jull et al. (2015).

Exposure age calculations

We calculated exposure ages (Table 1) using V3 of the online calculators formerly known as the CRONUS-Earth online calculators (Balco et al., 2008; https://hess.ess.washington.edu/). For these calculations, we used the standard atmosphere, a sample density of 2.7 g cm^{-3}, zero erosion rate, and the Tropical Andes

production rate calibration dataset for Huancane IIa moraines (Kelly et al., 2015). Exposure ages are calculated using the “St” scaling method of Stone (2000).

References (supplement-only):

Balco et al., 2008 - https://doi.org/10.1016/j.quageo.2007.12.001
Corbett et al. 2016 - https://doi.org/10.1016/j.quageo.2016.02.001
Jull et al., 2015 - https://doi.org/10.1016/j.quageo.2013.09.003
Kelly et al., 2015 - https://doi.org/10.1016/j.quageo.2013.10.007
Kohl and Nishiizumi, 1992 - https://doi.org/10.1016/0016-7037(92)90401-4
Nishiizumi et al., 2007 - https://doi.org/10.1016/j.nimb.2007.01.297
Stone 2000 - https://doi.org/10.1029/2000JB900181
Wilcken et al. 2022 - https://doi.org/10.5194/gchron-4-339-2022
